# A Cross-Sectional Survey Study About the Most Common Solitary and Social Flow Activities to Extend the Concept of Optimal Experience

**DOI:** 10.5964/ejop.v11i4.866

**Published:** 2015-11-27

**Authors:** Tímea Magyaródi, Attila Oláh

**Affiliations:** a Department of Personality and Health Psychology, Institute of Psychology, Eötvös Loránd University, Budapest, Hungary; Academy of Special Education, Warsaw, Poland

**Keywords:** solitary, social, flow, interactionism, demographic, induction, activity

## Abstract

Previous assumptions note that the most powerful experiences of engagement are shared with others. Therefore, in the framework of positive psychology, to expand the dynamic interactionism-related flow theory, we have attempted to conduct an exploratory study about flow to reveal the most common activities that can trigger this experience during solitary or social situations. The study involved 1,709 adult participants from Hungary (Age: M = 26.95, SD = 11.23). They read descriptions about optimal experience in solitary and social situations and were asked to identify the activity from their life that is most typically followed by the described experiences. The social context was supplemented by other flow-related questions for a deeper understanding and to contribute to the research. According to the results the most typical solitary flow activities are found to be work, sports, creative activities and reading. The most common flow-inducing social activities are work and sports. The choice of the most frequent flow-inducing activities in both solitary and interpersonal situations is dependent on the gender of the respondent, and various demographical factors can influence the frequency of flow experiences in different contexts. Analysis reveal that optimal experience during a social interaction is determined by the perceived level of challenges, the perceived level of cooperation, the immediateness and clarity of the feedback, and the level of the skill. Our study may contribute to the broadening purpose of positive psychology as it focuses on the interpersonal level in relation to flow experience, which, in turn, may also support a higher level of well-being.

## Introduction

Positive psychology, a science of the new millennium, aims to investigate positive experiences and personality factors that are embedded in social contexts (Seligman & Csikszentmihalyi, 2000). With regard to the effort on the part of positive psychology to not only establish research at the individual level, but also check the path towards well-being and growth at dyadic and group levels (Sheldon, Kashdan, & Steger, 2011), the main task of the present paper is to focus on the social level of positive experiences, highlighting Csikszentmihalyi’s flow concept (1990) in a social environment. When a person is totally involved in the perceived challenges of the activity at hand, his behavior becomes almost automatic as he enters the flow zone. The state of flow is universal and can be experienced in several situations (Csikszentmihalyi, 1990) in everyday life. Since Csikszentmihalyi presented his flow theory in 1975, several research studies were published dealing with different activities in which people can experience flow (e.g., flow at school: Rogatko, 2009; Shernoff, Csikszentmihalyi, Shneider, & Shernoff, 2003; at work: Bakker, 2008; Fullagar & Kelloway, 2009; in sports: Jackson, Thomas, Marsh, & Smethurst, 2001). Our work presents a description of the prevalence of activities that can induce flow in both solitary and social situations in everyday life, using a survey technique with a cross-sectional design, toward the exploratory study of the special flow-inducing acts and their characteristics in interpersonal contexts.

When someone is fully engaged in a challenging task, the basic conditions of being able to enter the flow zone are satisfied (Csikszentmihalyi, Abuhamdeh, & Nakamura, 2005). First, the individual should set clear, proximal goals (Nakamura & Csikszentmihalyi, 2002) that indicate the direction and aim of the activity. Second, this task needs to be challenging, so that it can be performed by someone with high skills, and there is a balance between the perceived challenges and perceived skills (Csikszentmihalyi et al., 2005). The third condition of flow is the presence of the clear and immediate feedback that addresses the changing environmental demands and the progress of the person in this context (Csikszentmihalyi et al., 2005). When a person is already in the flow zone, some characteristics can describe the dynamics of flow (optimal) experience (Nakamura & Csikszentmihalyi, 2002): intense and focused attention on the activity; loss of reflective self-consciousness; a sense of control over actions; distortion of temporal perception; and viewing of the activity as intrinsically rewarding. Therefore, one of the consequences of being in the flow zone during an activity is the desire to repeat it and find new challenges to overcome.

As noted earlier, research has shown that several types of activities can satisfy the conditions of flow (e.g., Rogatko, 2009; Shernoff et al., 2003). Csikszentmihalyi (1990) states that regardless of culture, social class, age, and gender, flow is described to have the same characteristics. Although the experience of flow may seem to be a universal phenomenon (Massimini & Delle Fave, 2000), some factors can vary the experience of flow in different groups. For instance, the findings of Heo, Lee, Pedersen, and McCormick (2010) suggest that retirement status and location can contribute to the flow experience of elderly people. According to Mannell, Zuzanek, and Larson (1988), Canadian retirees felt flow mostly during activities chosen freely by them.

Some studies that identify the most common flow-inducing activities (Csikszentmihalyi, 1998) mainly used experience-sampling methods (ESM, Hektner, Schmidt, & Csikszentmihalyi, 2007). It was found that among representative adults and teenagers, flow is common during work, studying, driving or transportation, talking, socializing, and sex, and it is the most typical quality of the experience while practicing hobbies, playing sports, and watching movies. Among leisure activities, games, sports, and other hobbies induced flow the most, according to U.S. teenagers (Csikszentmihalyi, 1998).

We should highlight the potential importance of a study that aims to reveal the most common flow-inducing activities, both in solitary and in social contexts, as mostly interview-based (Csikszentmihalyi & Csikszentmihalyi, 1988), and ESM-based studies have been carried out with regard to this question until now. In these ESM-based studies, participants were asked about the quality of the experiences in terms of the different flow and anti-flow channels (Csikszentmihalyi, 1990). The ESM approach is illustrated by Csikszentmihalyi and LeFevre’s (1989) study, in which a majority of adults reported flow experiences during work and not during leisure activities (in this research, the biggest components of leisure activities were watching television and reading).

Flow is said to be universal (Csikszentmihalyi, 1990) and we can also label it as being non-specific. The nature of the experience is the same, but the activity that can trigger it may differ among cultures, sub-cultures, groups, ages, or genders. It was found (Hektner et al., 2007) that managers and engineers experience flow during their work more often than other workers. From a cultural perspective, according to a study, U.S. adolescents have a more optimal experience while studying than their Italian peers (Hektner et al., 2007). Moreno Murcia, Cervelló Gimeno, and González-Cutre Coll (2008) found no meaningful differences between male and female adolescents in terms of general dispositional flow, but some results strengthen the hypothesis that activities which enhance flow may differ for males and females. For example, Csikszentmihalyi and Schneider (2000) found that girls achieve higher levels of flow during classroom activities than boys. It was revealed that women report more micro-flow experiences (Allison & Duncan, 1988) in everyday activities.

In conclusion, flow experience has been studied mainly in solitary situations, as it is a subjective state of being completely involved in an activity (Csikszentmihalyi et al., 2005). Flow theory is strongly related to the concept of dynamic interactionism (Magnusson & Stattin, 1998), as the behavior is the result of persistent and bilateral interaction between the individual and the situation (Reynolds et al., 2010). During the experience, the person and the context compose a dynamic system (Nakamura & Csikszentmihalyi, 2002), as the person tackles the challenges raised by the task in the environment, improving skills supported by feedback, and going toward the goal and reaching the optimal level of experience. The experience of flow is supported by emerging motivation as, during the activity, a growing level of challenges meeting a growing level of skills. This results in open-ended rewards that enable the person to find more challenges in order to stay in the zone (Csikszentmihalyi & Rathunde, 1993). There are several studies using ESM and interview techniques (Csikszentmihalyi & Csikszentmihalyi, 1988) that strengthen the idea that social activities can also be the source of flow activities (Hektner et al., 2007). During these interpersonal activities, the other person, as part of the environment, can support the maintenance of flow. Csikszentmihalyi and Csikszentmihalyi (1988) propose the concept of shared flow, a phenomenon in which the other person can be the source of the challenge or the feedback about the performance. There have been several remarkable hypothetical suggestions that describe the common flow experience, but these have not yet been confirmed by evidence-based studies (shared flow by Csikszentmihalyi & Csikszentmihalyi, 1988; relational flow by Moore, Drake, Tschannen-Moran, Campone, & Kauffman, 2005; group flow by Sawyer, 2007; social flow by Walker, 2010; networked flow by Gaggioli, Milani, Mazzoni, & Riva, 2011). Csikszentmihalyi (1998) observes that talking, socializing, and sex can also be sources of optimal experience. Therefore, it is important to identify the various types of interpersonal activities that can support flow and its synchronization and then to understand the characteristics of these experiences. In this paper, we aim to present the most common flow-inducing activities in solitary and social situations, as reported by the subjects. Previously in flow research, social contexts were studied as just one source of the optimal experience; the focus was mainly on the individuals (Nakamura & Csikszentmihalyi, 2002) while extended questions were not analyzed. The use of the survey approach in flow research is appropriate when the aim is the measurement of flow dimensions or the exploration of differences in the occurrence of optimal experience between situations (Nakamura & Csikszentmihalyi, 2002). As our main goal was to identify the most typical solitary and social flow activities, to measure the effect of demographic variables such as age, gender, and educational level on the frequency of the experienced flow in different contexts, and to compare our conclusions with the results of previous research, we carried out an exploratory cross-sectional study using the survey method on a bigger sample. Another aim of this study is to broaden flow research with a social perspective by highlighting the special characteristics of the most frequently reported flow-inducing activities in interpersonal situations.

## Method

### Participants

A total of 1709 adult participants (*N_female_* = 1114; *N_male_* = 595) took part in the study. They were recruited through a non-probability convenient sampling procedure. Participation was voluntary and anonymous, no incentives were offered to the subjects.

The mean age of the sample was 26.95 (*SD* = 11.23). Table 1 specifies the demographic characteristics of the participants.

**Table 1:** Demographic Characteristics of the Participants (N = 1709)

Characteristic	%
Gender	
Male	34.80
Female	65.20
Residence	
Capital city	38.60
City, town	22.60
Village	27.70
Abroad	11.00
Relationship status	
Single	44.10
In a relationship	55.90
Education	
Elementary	3.60
Secondary	63.70
Higher	32.80

### Materials

After answering the demographic questions (gender, age, residence, relationship status, and education level), participants were asked to fill in an online questionnaire about their flow experiences in a solitary and a social context.

#### General flow description (GFD)

This item was used to introduce the participants to the nature of flow. This material was based on Jackson and Roberts’ (1992) general flow index. The description reads:

People often report that they are going through a positive experience, when their attention to the task ahead of them is totally engaged and they are completely preoccupied in what they're doing. In a moment like this, they are just concentrating on the activity, which is being done by themselves. In this case, they feel they cannot escape from the actual task, and they may forget about eating or other obligations. They are so involved that other things are ignored; other feelings, problems, and tasks are forgotten. However, they are progressing well in the activity, they do not experience fatigue, and they do not think that the activity is demanding. The time spent doing the task passes in an almost unnoticed way. They know exactly what their purpose is and they progress towards their goals. Looking back on the experience, they can feel that they have developed themselves during the activity. Activities like these are good to do just for the sake of the experience.

After reading the description, the participants answered questions about the frequency of the described experience in their everyday life, on a five-point Likert scale (from “not at all” to “a great extent”). Then the most typical activity was identified by each participant along with its duration in hours.

The description was checked on a smaller sample (*N* = 20) before its use to make sure about its understandability and validity.

#### General flow description in social interactions (GFSD)

Based on the method of the GFD, we created similar documentation about the common flow experience during social interactions, supported by assumptions in the existing literature (e.g., Csikszentmihalyi & Csikszentmihalyi, 1988; Walker, 2010). The description is as follows:

People often report that they have a positive experience when they are working together on a task with someone. Their attention is taken up by the common task and they are totally preoccupied by the activity at hand. They are just concentrating on what they are doing. During this time, they feel that they cannot break away from the action; they forget to eat and perform other obligations. Though they are progressing quite well, they do not feel tired, nor do they perceive the activity as demanding. Both partners play a role to meet the challenges ahead. They know the exact purpose and they have a common strategy to achieve this goal. The partners help each other, integrate with consistency, motivate themselves, and learn from each other. Looking back on the experience, they realize how much they have developed during the activity and how they affected each other’s performance positively.

After reading the description, the participants answered a question about the frequency of the described experience in their everyday life, on a five-point Likert scale (from “not at all” to “a great extent”). Then the most typical activity was identified by the participant along with its duration in hours. To get more information about the flow experience in social contexts, the participants were asked to evaluate the activities on a five-point Likert scale (from “not at all” to “a great extent”) according to the perceived level of the challenge, their perceived skills, the clarity of the aim, and the continuity of the feedback. After evaluating the components of their flow experience, we asked the subjects to judge if the activity involved cooperation or competition, the level of which they had to evaluate on a five-point Likert scale (from “not at all” to “a great extent”).

This description was also checked on a smaller sample (*N* = 20) before its use to test its understandability and validity.

#### Flow State Questionnaire (PPL-FSQ)

The Flow State Questionnaire (PPL-FSQ) (Magyaródi, Nagy, Soltész, Mózes, & Oláh, 2013) aims to measure the basic dimensions of flow experience through 20 statements that have to be rated on a five-point Likert scale (from "strongly disagree" to "strongly agree"). The PPL-FSQ has two scales. One of them (C-S) measures the balance between challenges and skills with 11 items. This scale refers to the basic conditions to get into the flow zone. The other one (A) measures the absorption in the activity with 9 items. This scale represents the inherent characteristics of the flow experience. The internal consistency of the scales is psychometrically acceptable (Cronbach's α_C-S_ = .92; Cronbach's α_A_ = .91).

The descriptive statistics, reliability scores and intercorrelations of the psychological inventories used in this study are described in Table 2.

**Table 2:** Descriptive Statistics of the Used Variables With Intercorrelations

Scales	1.	2.	3.	4.	5.	6.	7.	8.	9.	10.	11.	12.	13.
1. GFD: frequency of flow	-												
2. GFD: duration of the experience (hours)	.08**	-											
3. GFSD: frequency	.43**	.06*	-										
4. GFSD: level of perceived challenges	.21**	.02	.30**	-									
5. GFSD: level of perceived skills	.12**	.01	.23**	.23**	-								
6. GFSD: clear goal	.11**	-.02	.24**	.28**	.44**	-							
7. GFSD: immediate feedback	.09**	-.04	.24**	.22**	.36**	.40**	-						
8. GFSD: level of cooperation	.11**	-.01	.26**	.22**	.32**	.37**	.30**	-					
9. GFSD: level of competing	.07**	.05*	.04	.18**	-.01	-.01	.02	-.23**	-				
10. GFSD: duration of the experience (hours)	.05	.24**	.16**	.09**	.04	.03	.02	.06*	-.06*	-			
11. PPL-FSQ: balance between challenges and skills	.13**	-.01	.21**	.04	.51**	.33**	.28**	.22**	-.07**	.02	-		
12. PPL-FSQ: Absorption in the task	.25**	.01	.31**	.29**	.26**	.28**	.31**	.26**	.08**	.033	.36**	-	
13. PPL-FSQ: total score	.23**	.00	.31**	.19**	.47**	.37**	.36**	.29**	-.00	.03	.85**	.80**	-
*N*	1709	1536	1709	1709	1709	1709	1709	1709	1709	1458	1709	1709	1709
*M*	3.47	2.84	3.19	3.62	3.99	4.29	3.71	4.3	2.36	2.53	3.76	3.40	7.16
*SD*	.86	2.63	.94	.97	.79	.84	.94	.82	1.17	2.68	.5	.43	.76
α	-	-	-	-	-	-	-	-	-	-	.84	.84	.87

*Note.* GFD = General Flow Description; GFSD = General Flow Description in Social Interactions; PPL-FSQ = Flow State Questionnaire.

*p < .05. **p < .01.

### Procedure

The Ethical Committee of the Eötvös Loránd University has supported the research. Participants were recruited through university courses and social media sites. Participants had to be at least 18 years of age and free from any psychiatric or neurological problems. Subjects were asked to fill in an online questionnaire after they gave their informed consent.

## Results

**Figure 1:**
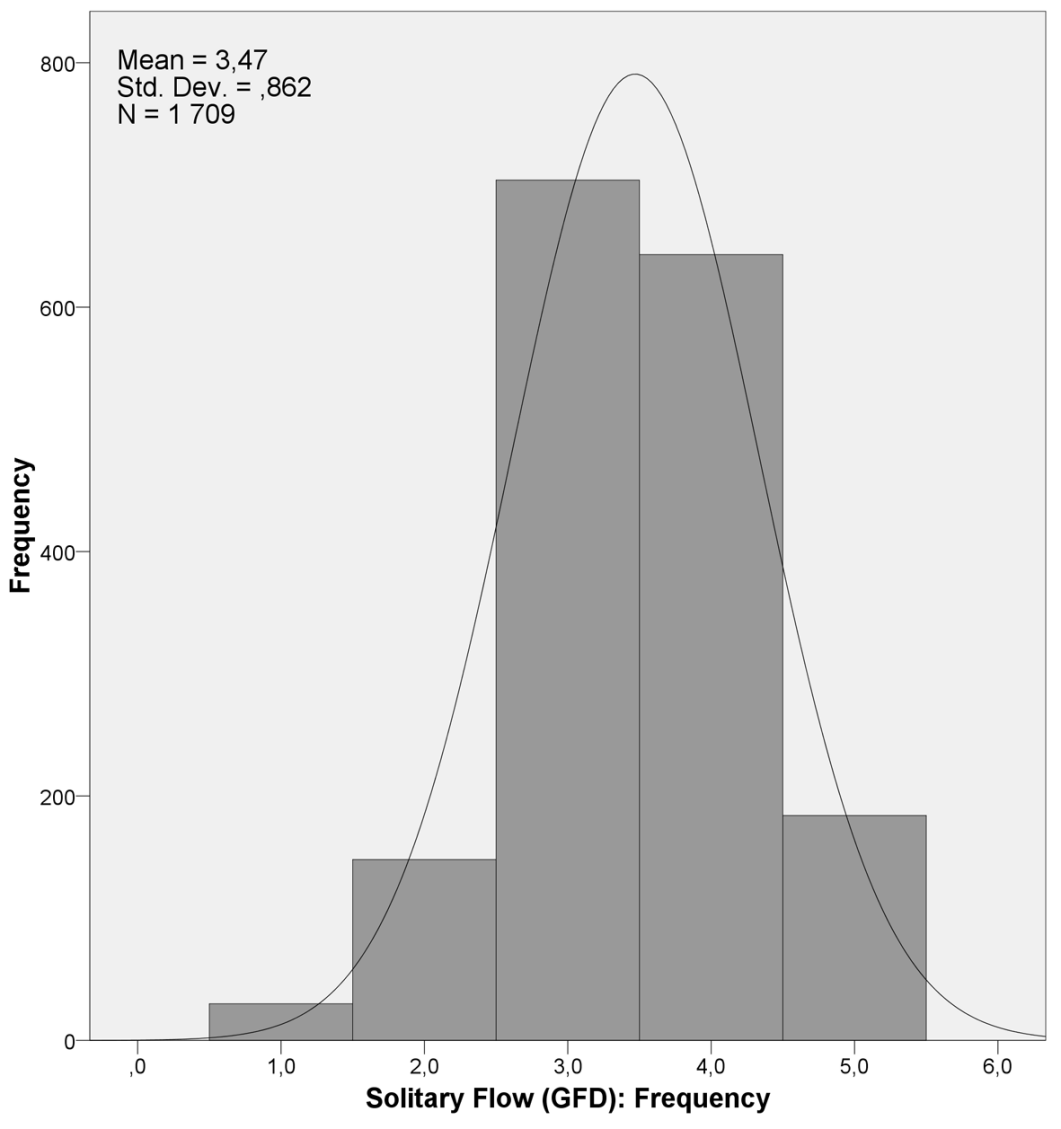
Histogram of the frequency of solitary flow experience variable.

We first provide an analysis about the overall study of the data with regard to the gender differences and demographic variables related to flow in solitary situations. Figure 1 illustrates the distribution of the responses of the flow frequency variable during solitary activities.

According to the demographical analysis, the t-test of independent samples shows significant difference between male and female groups with regard to solitary flow experiences, *t*(1534) = 2.08, *p* < .05. Men spend significantly more time working an activity in flow. We find no gender difference regarding the frequency of flow in individual activities.

We have transformed the classifying variables (gender, education level, residency, and relationship status) into dummy variables. Then, with the addition of the age variable, we have run a linear regression analysis using a stepwise method to identify the demographic factors that can predict the general frequency of solitary flow. In the model, it is the higher education degree, β = 0.06, *t*(1707) = 2.48, *p* < .05, that significantly predicts the general frequency of flow in solitary situations, *R^2^* = 0.004, *F*(1,1707) = 6.13, *p* < .01.

The subjects, by responding to an open-ended question, have indicated their most common solitary activity that induces flow. We find several labels for the most common flow-inducing activities, which we have classified into different categories based on their contents. Two researchers have individually made the classification, following which the inter-rater reliability of the classification has been checked. There are no discrepancies related to the content of the categories. There are 16 categories and an additional category for the answers of those subjects who were not familiar with the given description of flow. As Table 3 illustrates, 1,661 answers have been interpreted into categories, leaving 48 missing answers. The frequency results show that the majority of flow activities are experienced during work, reading, sports, and creative activities. All of these are above 10%, making a total of 71.80% altogether (*N* = 1266).

**Table 3:** The Frequencies of the Mentioned Solitary Flow Activities in the Whole Sample

Solitary Flow Activity	%
Work/studying/task solving	31.70
Reading	14.00
Sport	13.50
Creative activities	12.60
Housework, work in the garden	6.40
Music	5.20
Leisure activities	5.10
Dance	2.20
Communication, chatting	1.90
Playing games	1.40
Organizing tasks	1.20
Playing video games	1.10
Acting	0.70
Dealing with the computer	0.70
Love life	0.60
Driving	0.30
No experience like this	1.30

As the four most frequent flow activities are analyzed (*N* = 1266), significant differences can be found in terms of both the frequency of flow, *F*(3,1262) = 3.17, *p* < .05, and the duration spent in flow, *F*(3,1144) = 19.10, *p* < .001. According to the LSD post hoc tests, work situations are mentioned as significantly more frequent solitary flow-inducing situations than reading activities (*p* = .02). Creative activities are mentioned as more frequently flow-inducing activities than sports (*p* = .05) and reading (*p* = .02). Regarding the duration of the time spent in flow in the different activities, we can highlight, based on the results of LSD post hoc tests, that flow is longer during work activities, compared to sports, creative activities, and reading (*p* < .05). To evaluate the gender differences in the typical individual flow activities, we ran a chi square test, the results of which suggest the type of flow activities that are dependent on gender, χ^2^ = 30.99; *df* = 3; *p* < .001. However, this relationship is weak, φ = 0.16, *p* < .001.

As this study has been executed with an explorative focus, in order to reveal the specific relationship between the demographic variables and the various frequently flow-inducing activities in an individual context, binary logistic regression analysis has been carried out by a forward stepwise method. Our aim was to predict the most common solitary flow-inducing activities using the variables of gender and educational level (dummy variables: elementary, secondary and higher education), as well as the interaction between gender, education level, and age.

Whether work, studying, or task-solving is the most common flow-inducing solitary situation can be predicted by gender, education level, and age, as Table 4 illustrates. Women, higher education degree-holders, and older participants tend to choose these activities with more probability than men, subjects who do not have a higher education degree, and younger participants. Additionally, the interaction between the elementary education level and gender has lower probability to choose work, study or task-solving situations as flow-inducing in individual context.

**Table 4:** Binary Logistic Regression About the Predictive Variables Related to the Probability of Experiencing Flow in Different Individual Activities.

Dependent Variable	Independent Variable	*p*	Exp(B)	95% CI	Model			
					Neagelkerke *R Square*	χ^2^	*df*	*p*
Work, studying or task-solving					0.06	53.17	4	.00
	Gender (1)	.00	1.61	[1.26, 2.04]				
	Higher education	.04	1.32	[1.01, 1.72]				
	Elementary education * Gender (1)	.02	0.16	[0.04, 0.70]				
	Age	.00	1.02	[1.01, 1.03]				
Sport					0.04	35.01	4	.00
	Gender (1)	.02	1.42	[1.06, 1.91]				
	Elementary education	.02	2.17	[1.15, 4.10]				
	Higher education	.02	0.64	[0.44, 0.92]				
	Age	.02	0.98	[0.96, 1.00]				
Creative activities					0.01	8.36	1	.004
	Gender (1) * Secondary education	.01	0.56	[0.37, 0.85]				
Reading					0.04	28.86	3	.00
	Gender (1)	.00	0.45	[0.32, 0.63]				
	Gender (1) * Elementary education	.04	3.23	[1.07, 9.75]				
	Age	.04	0.99	[0.97, 1.00]				

*Note.* Exp(B) = the change of the odds ratio. CI = Confidence Interval.

Gender and elementary education provide higher odds for the choice of sports. Women, as well as people who have completed elementary studies, tend to choose this kind of activity, though a higher education level and age lower the odds for this choice.

The choice of creative activities as flow sources can be affected by the interaction of gender and completed secondary studies; in case of such interaction, the odds for this choice become lower.

Women and younger participants tend to choose reading more than men and older participants. The interaction of gender and elementary education seems to augment the odds for this choice.

**Figure 2:**
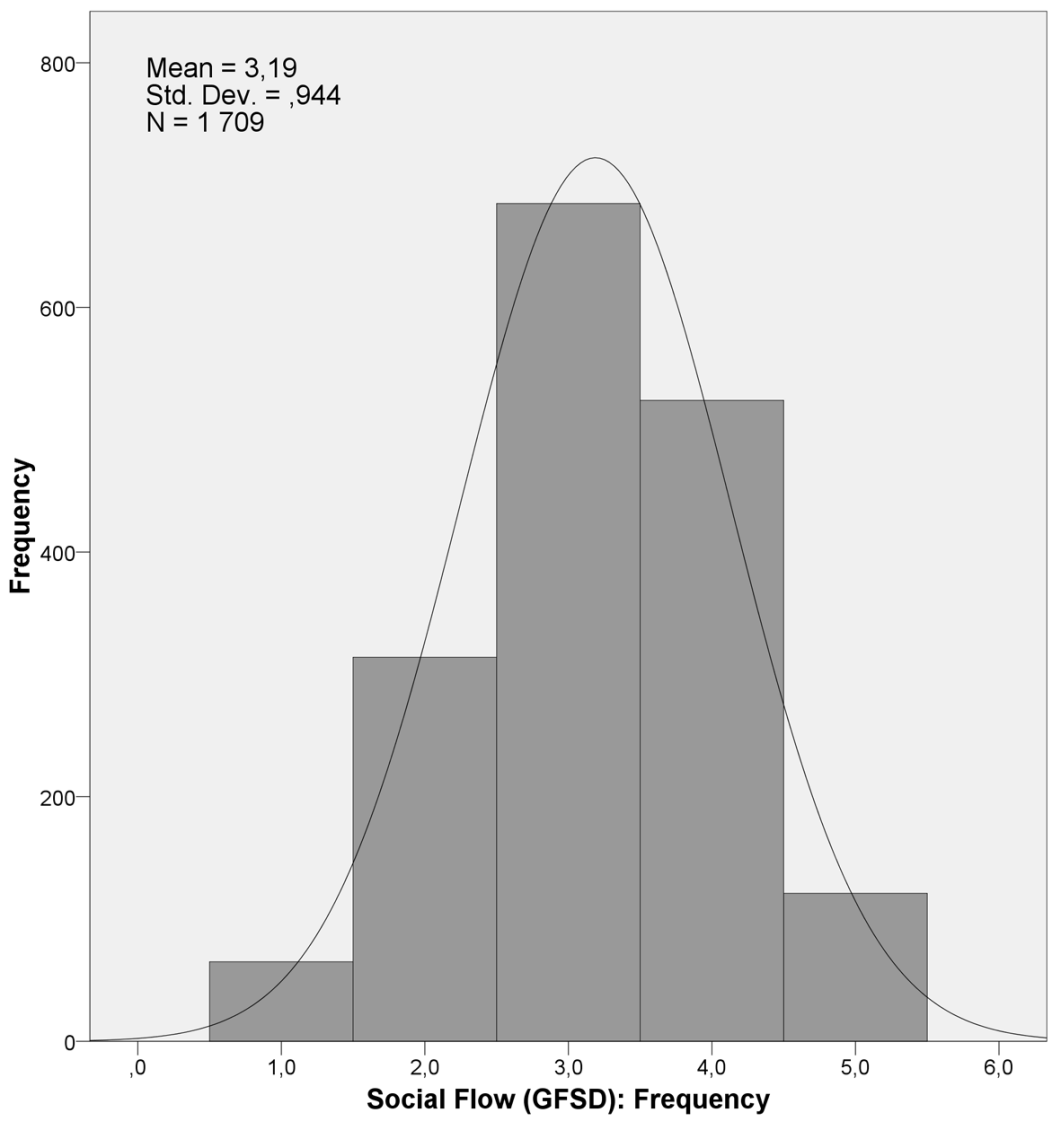
Histogram of the Frequency of Flow Experience in Social Activities Variable.

With regard to analysis of the interpersonal flow contexts, distribution of the responses of the flow in a social context is presented by a histogram (Figure 2).

With regard to the results on gender differences, we observe that in the case of the female subjects, the clarity of goals, *t*(1707) = −3.90, *p* < .001, the immediateness of the feedback, *t*(1707) = −3.93, *p* < .001, and the level of cooperation, *t*(1707) = −4.21, *p* < .001, during social flow activities are significantly higher than in the case of male subjects, though the level of competition for males during social flow activities is significantly higher, *t*(1707) = 5.62, *p* < .001. Gender differences also exist in the level of total flow experience, *t*(1707) = −2.27, *p* < .05, during these situations. The balance of the perceived challenges and skills, *t*(1707) = −2.04, *p* < .05, and the level of absorption in the task, *t*(1707) = −2.62, *p* < .01, are also significantly higher for women.

According to the linear regression analysis, one of the demographical variables, namely age, is a significant predictor for the frequency of social flow experiences. Age, β = 0.06, *t*(1707) = 2.33, *p* < .05, predicts the higher frequency of flow experiences during social activities, *R^2^* = 0.003, *F*(1,1707) = 5.41, *p* < .05.

The subjects have responded to an open-ended question to indicate their most common social activity that induces flow. As Table 5 illustrates, the documented contexts of social flow experiences have been classified by the authors through a judgment procedure—for instance, with a qualitative content analysis in the case of solitary activities. There are 14 categories and an additional category for the answers of those subjects who were unfamiliar with the given description of social flow and flow synchronization. A total of 1691 answers have been distributed into categories, while 18 answers are missing.

**Table 5:** The Frequencies of the Mentioned Social Flow Activities in the Whole Sample

Social Flow Activity	%
Work/studying/task solving	44.40
Sport	15.20
Housework, work in the garden	6.70
Leisure activities	5.50
Playing music together	4.30
Organizing tasks	4.10
Communication, chatting	3.50
Creative activities	3.20
Dance	2.90
Playing games	2.50
Love life	2.30
Acting (theater)	1.70
Playing video games	1.20
Reading together	0.40
No experience like this	2.20

The results show that the vast majority of social flow activities are experienced during work and sports activities. All of these are above 10%, making a total of 59.60% altogether (*N* = 1168).

Analysis of the two most frequent social flow activities – i.e., work and sports (*N* = 1168) – shows that there are significant differences between them. During involvement in sports, the perceived level of challenge, *t*(1166) = −2.81, *p* < .01, the level of competition, *t*(1166) = −12.41, *p* < .001, and the absorption in the task *t*(1166) = −3.47, *p* < .001, are significantly higher than in the cases of working, studying, and task-solving situations. During work, the perceived level of the skills, *t*(1166) = 2.09, *p* < .05, the level of cooperation, *t*(1166) = 4.39, *p* < .001, and the perceived duration, *t*(1014) = 3.45, *p* < .001, are significantly higher than during sport activities.

To test the gender differences in the typical flow activities, we have run a chi square test, the results of which suggest that the type of flow activities is dependent on gender, χ^2^(3) = 7.24; *p* < .05; however, this relationship is weak, φ = −0.08, *p* < .01, as in the case of choosing the typical solitary flow activities.

In order to find the specific relationship between the demographic variables and the various frequently flow-inducing activities in social interaction, binary logistic regression analysis has been carried out by a forward stepwise method. Our aim was to predict the most common flow-inducing activities in social contexts, using gender, educational level (dummy variables: elementary, secondary, and higher education), relationship status (dummy variables: single and in a relationship), as well as the interaction between gender, education level, and age.

Results of the binary logistic regression analysis (Table 6) show that there is no significant relationship between the choice of the activities and relationship status. In case of gender, the odds of choosing work, study or task-solving as a flow-inducing activity in a social context are lower in case of female subjects. Another significant predictor of this dependent variable is having a higher education level, while age seems to augment the probability of choosing these kinds of activities for flow induction in a social situation.

**Table 6:** Binary Logistic Regression About the Predictive Variables Related to the Probability of Experiencing Flow in Different Social Activities

Dependent Variable	Independent Variable	*p*	Exp(B)	95% CI	Model			
					Neagelkerke *R Square*	χ^2^	*df*	*p*
Work, studying or task-solving					0.06	45.62	3	.00
	Gender (1)	.01	0.68	[0.51, 0.90]				
	Higher education	.00	2.01	[1.37, 2.94]				
	Age	.01	1.02	[1.01, 1.04]				
Sport					0.06	45.62	3	.00
	Gender (1)	.01	1.48	[1.11, 1.98]				
	Higher education	.00	0.50	[0.34, 0.73]				
	Age	.01	0.98	[0.96, 0.10]				

*Note.* Exp(B) = the change of the odds ratio. CI = Confidence Interval.

Gender is a significant predictor in the case of choosing sports as the most frequent flow-inducing activity; women choose sport with a higher probability for finding optimal experience. Higher education and age lower the odds of choosing sport activities.

To establish a prediction model that can explain the frequency of social flow activities, we have run a linear regression analysis using a stepwise method. The question is whether the flow conditions – i.e., the perceived level of the challenges and skills, the clarity of the aim, and the clarity and immediacy of the feedback (Nakamura & Csikszentmihalyi, 2002), and the level of cooperation or competition – contribute to a higher frequency of flow during common social interactions. According to the result, a predictive model can be formed out of four components, *R^2^* = 0.15, *F*(4,1704) = 76.30, *p* < .001. The predictive variables are the following: the perceived level of challenges, β = 0.22, *t*(1704) = 9.304, *p* < .001, the level of cooperation, β = 0.14, *t*(1704) = 5.94, *p* < .001, the immediacy and clarity of the feedback, β = 0.12, *t*(1704) = 4.66, *p* < .001, and the perceived level of skills, β = 0.10, *t*(1704) = 3.89, *p* < .001.

## Discussion

This investigation aims to explore the activities that can induce flow in both solitary and interpersonal situations most frequently, and to extend the knowledge about optimal experience during common social interactions, in order to continue studying this innovative field in positive psychology. Our research aims to broaden the designs of earlier research, which mainly used the ESM method, to investigate a broader sample to reveal the most typical flow-inducing activities in social interactions, along with their flow-related and demographical associations. In this study, we have used a direct survey technique to ask about the most common contexts in which flow experience can be observed as a subjective state during a solitary task and in social interactions.

At first, we measured the most common situations that can induce flow without any social interaction. According to the original model of flow (Csikszentmihalyi, 1975), these experiences can differ from each other based on their complexity, which has its roots in the level of the perceived balance of the challenges and skills of the person (Moneta, 2012). Less complex activities are labeled as micro-flow experiences, while the more complex ones are macro-flow experiences. Micro-flow experiences mainly occur in and add meaning to daily life (Csikszentmihalyi, 1975), while deep flow or macro-flow is less frequent. There is a significant difference between male and female subjects in this regard; men reported a longer duration of solitary flow experiences than women. Another task is to investigate the factors that may promote these longer flow experiences. According to a review about gender differences (Block, Zakay, & Hancock, 1999), several variables influence the differences in time judgment by men and women. As time gets distorted during flow, this result must be interpreted carefully. There is an altered sense of time (Csikszentmihalyi et al., 2005) during an optimal experience. Therefore, this result may give us information either about the gender differences in time judgment during this state or about the perception of the flow experience itself. For example, women are more likely to experience micro-flow situations (Allison & Duncan, 1988) than men.

In terms of the demographical analysis, we find that higher education can predict the general frequency of flow in solitary situations, though the explanatory power is moderate; it can predict 0.4% of the variances of flow frequency. As we have quoted before, Csikszentmihalyi and LeFevre (1989) found that managers experience flow mainly during their work, while clerks and blue-collar workers felt this optimal experience during their leisure activities. Our result may strengthen this finding. Higher education can possibly predict more complex and multifaceted tasks at work that can fulfill the requirements of flow (Nakamura & Csikszentmihalyi, 2002).

The classification and distribution of solitary flow experiences emphasizes four typical flow-inducing activities: work, study, task-solving, and leisure activities such as reading, sports, and creative activities. Csikszentmihalyi (1990) emphasizes mainly work, sports, and leisure activities as flow-inducing. It is really important to note that the source of the flow theory lies in the study of creative people (Csikszentmihalyi, 1975) who recognize this kind of optimal experience during their creative activities. However, according to our results, this kind of task can be found in people’s everyday lives. These activities took up almost 72% of the sample results. According to the results, we can find the main difference between these situations in terms of frequency of flow and the perceived duration of the experience during the activity (Csikszentmihalyi & LeFevre, 1989). Work, study, and task-solving situations differ from the three other categories, as they induce the longest flow experience according to the evaluation of the subjects. Sporting activities take the least time among the four types of activities. At the workplace, during the school day, or in a task-solving situation, there is a wide range of time that can be used, and as work is the most common context in which people experience flow, it also offers the widest range of time.

Specific demographic variables relate to the choice of the most typical flow activity in the case of solitary situations. Women and those who have higher education choose work as a flow-inducing activity. Age also has a significant influence; if someone is older, the choice of work situations is more likely. The interaction between elementary studies and gender has a lower effect on the probability of choosing work as a flow-inducing activity. Therefore, the effect of gender cannot be interpreted in itself, but only in terms of the educational level, though the elementary group is the smallest education group in the sample (*N* = 61; *N_women_* = 22, *N_men_* = 39). Women and people who have completed elementary studies tend to choose sport as a source of flow experience, but a higher education level and age lower the probability for this choice. The choice of creative activities as flow sources can be affected by the interaction of gender and completed secondary studies; in case of such an interaction the odds for this choice are lower. Women and younger participants tend to choose reading with greater probability than men and older participants. The interaction of gender and elementary education seems to augment the odds for this choice. We can surmise from these results that flow can be experienced independent of gender, education level, or culture (Csikszentmihalyi, 1990), but the situations that may induce the experience can differ. Earlier results suggest the frequency and quality of optimal experiences may vary at the individual level (Collins, Sarkisian, & Winner, 2009). Studies about the effect of age on flow (Lawton, 1989) suggest that older adults may experience flow if the perceived challenges are slightly above the skills of the person. The probability of experiencing micro-flow experiences (Csikszentmihalyi, 1975) is higher; therefore, the possibility of experiencing flow is also greater.

Research focusing on the prevalence of flow in activities in a social interaction has not been carried out yet, as previous studies have focused on flow as subjective states without describing the common context or the working mechanism of the shared experience. In terms of the gender differences, female subjects report higher levels of goal clarity, more immediate feedback, and higher level of cooperation than men; therefore, they can experience the flow components at a higher level, with a higher quality of experience (the main conditions for flow and the absorption in the task are significantly more satisfied in case of the female sample than the male one). Previous studies have focused on gender differences in goal orientation, and results consistently show that women have higher goal orientation than men (Baser, Bayar, & Ghorbanzadeh, 2013). These results agree with the classification of Moreno Murcia et al. (2008) about the different gender preferences of task- and ego-oriented climates. Regarding the special characteristics of flow in social contexts, men perceive a higher level of competition during these flow-inducing social activities. Out of the demographic variables, age predicts flow frequency in social situations. Cornwell (2011) shows that older people spend less time actively in their personal networks, though our sample has a mean age of 26.95 years (*SD* = 11.23), people in the young adulthood phase in terms of the psychosocial theory of Erikson, when partners in friendship, sex, competition, and cooperation are in the focus of connectedness of the person (Karcher & Benne, 2008). The age distribution related to this particular phase of life may explain the influence of age in this context.

Csikszentmihalyi (1990) proposed that the most intense and meaningful experiences in people’s life are the results of the relationships with their families. Graham’s (2008) study suggests that in close relationships, the activity level affects the quality of flow which, in turn, can heighten the level of satisfaction with the relationship; thus, these factors may strengthen each other. According to the results, flow in social activities is most frequent during work and sports activities; about two-thirds of the sample gave answers that fall in these categories (59.6%). It is notable that in both solitary and social contexts, work is the activity most frequently given in response. This can be linked to the paradox of work (Csikszentmihalyi, 1990). As work, studying, and task solving are good contexts to experience the ingredients of flow, these kinds of activities can provide major challenges that require development of skills. People report flow mostly during work situations where there are clear goals and probably immediate and clear feedback as well. In previous studies (Moore et al., 2005; Sawyer, 2007; Walker, 2010), work or common learning situations and sport situations were considered as the sources of the common flow experience; consequently, our result may contribute to these earlier hypotheses. In this study, sport activities are accompanied by a higher level of the perceived challenges and competition, and the absorption in the task is also higher. However, during work, study, or task solving, the level of the perceived skills and cooperation is higher and the perceived duration of the experience is longer compared to sport activities. This finding is in accordance with the notion of ego- and task-oriented activities (Moreno Murcia et al., 2008) that labels sport as a competitive, ego-oriented climate while work and studying are mastery- and task-oriented climates. We might consider the note by Jackson and Csikszentmihalyi (1999), which claims that since sports is about testing the physical body, it makes these test conditions progressively difficult; thus, people who play some kind of sports can often meet challenging situations. Different factors may contribute to a higher flow experience in different situations, in accordance with the previous findings. Our data supports the earlier data (Jackson et al., 2001) about the most critical components of flow in sports: it was found that the perceived skill component of the challenge-skill balance is a key factor in optimal sport experiences, while our result emphasizes that the higher level of perceived challenges and the factor of competition is also critical.

Several papers discuss the flow in social situations, but there has not yet been evidence-based consistency about the type of activities regarding their cooperative or competitive aspects. According to our results, the vast majority of the reported social flow activities are cooperative situations (86.7%) and the perceived duration and the level of the balance between the perceived challenges and skills in these cooperative tasks are significantly higher in cooperative situations. This finding corresponds with Walker’s (2010) assumption about social flow, as he suggested that in sport activities, which are the sources of this kind of common experience, people are interdependent and there is a high level of cooperation. According to previous findings, synchronization, which can make psychological and physiological reactions more intense (e.g. Ackerman & Bargh, 2010), is more frequent during cooperative activities than during competitive ones (Delaherche et al., 2012).

In the case of social flow experiences, there is a slight effect of gender on the choice of the social activities. Men choose work, study or task solving as social flow inducing-situations while women are more likely to choose sports. From another perspective, this finding refers to the preferences of different orientation types (Moreno Murcia et al., 2008); boys perceive a competitive or ego-oriented motivational climate while girls favor mastery- or task-oriented situations. The authors note that the significant others play the key role—if they believe that winning and demonstration of physical ability in competition with others defines success, ego-oriented climate will be promoted (for example sports), but if they feel that the most important indications of ability are effort and improvement, then this attitude supports a task-oriented climate (for example, work). Our results support this hypothesis, as sport in social flow situations is more competitive than work, while work, studying, and task solving are more cooperative than sports. Regarding the other demographic factors, higher education level can predict work as a choice of flow source in social contexts, while during sport activities higher education and age predict its odds in an opposite way: It is probable that younger people with less education choose sport as a flow-inducing common activity.

We aim to establish a prediction model that can explain the frequency of social flow activities through the flow conditions (the perceived levels of challenges and skills, the clarity of the aim, and the clarity and immediateness of the feedback) and the level of cooperative and competitive characteristics. In our results, the regression model explains the variance of the frequency of social flow activities and flow synchronization through the following four variables to a moderate degree (about 15%): the level of the perceived challenges, the level of cooperation, the immediateness and clarity of the feedback, and the perceived level of skills. Except for the perceived level of skills and the clarity of the aim, every other flow condition (Csikszentmihalyi et al., 2005) is in the model, so we may assume that the most important factors for a common flow experience are a high level of the perceived challenges (and here we note that these challenges may be set by the aim and direction of the activity, or perhaps the other person in the situation might be the source of the challenge—see the hypothesis of shared flow developed by Csikszentmihalyi & Csikszentmihalyi, 1988) with appropriate feedback about the progress, in the case of a cooperative task, as assumed by Walker (2010).

The conclusions that can be drawn from this exploratory study can be encouraging in the field of social flow experiences. However, there are several limitations to be considered; therefore, the results should be treated with caution. Regarding the choice of the method, as self-administration can lead to a bias, in future studies the use of a non-survey technique—for example, an ESM or interview method—should be used in order to reveal the specific occurrence and distribution of the daily experiences, but with the highlighted aim of focusing on flow-inducing social activities. The retrospective nature of the questions may have led the respondents into social desirability or retrospective bias, and although this approach is frequent in the literature (Moneta, 2012; Nakamura & Csikszentmihalyi, 2002) where methodology of flow measurement is highlighted, the recall of the memories based on a description can be distorted. This problem may be corrected if we can establish a real-time data administration with the ESM technique. Regarding the sampling, the representative nature of the sample was not ensured; therefore, the results have to be considered cautiously and the gender and other demographical variables need to be more even in future studies. As this study examined a Hungarian sample, we should consider generalizability of the results carefully. For future research, the possible cultural differences in specific flow-inducing activities may be interesting. Though we have discovered some connection between the demographic variables and the probable choice of flow-inducing activities, further studies should use a sample with a broader age range and more even distribution of demographic variables.

During the documenting of the GFD and GFSD, we were intent on making full and understandable descriptions about the experiences, but even though their usability was checked during a pilot study, a different measuring tool might have been better. Although the PPL-FSQ (Magyaródi et al., 2013) has acceptable internal consistency, since this questionnaire and the two other descriptive instruments are relatively new, each has to be refined in future works.

In future works, we aim to conceptualize the mechanism of flow experience in a common task accompanied by social interaction. Our future goal is also to clarify the different factors and personal characteristics that may contribute to the higher frequency of flow experience in interpersonal tasks by understanding its assumed accompanying mechanism (Nakamura & Csikszentmihalyi, 2002), which we labeled as flow synchronization. Through expanding the dynamic system of the person and context with the factor of another person as part of the environment, the mechanism of a new system may be understandable during the common flow experience. The effect of another person on the experience as well as the significance and quality of the relationship and the role of the other person might be revealed by conceptualizing flow synchronization. In future studies, flow research can be broadened with experimental design that reveals the specific causal relationship between flow experience and its possible causes and accompanying factors during a social interaction.

As noted in the beginning of the paper, it is an ongoing trend in positive psychology to conceptualize hypothesized phenomena that can contribute to a person’s mental growth, and then to operationalize it with an instrument in order to conduct evidence-based studies, on personal as well as dyadic or more complex social levels.

In conclusion, this work about flow in social interactions and its presumed mechanism—i.e. flow synchronization—aims to contribute to study those factors that, in accordance to Fredrickson’s (2004) broaden-and-build theory, can help people broaden their optimal experiences and build their own level of well-being.
